# Of mutualism and migration: will interactions with novel ericoid mycorrhizal communities help or hinder northward *Rhododendron* range shifts?

**DOI:** 10.1007/s00442-021-05081-9

**Published:** 2022-01-02

**Authors:** Taryn L. Mueller, Elena Karlsen-Ayala, David A. Moeller, Jesse Bellemare

**Affiliations:** 1 Department of Ecology, Evolution, and Behavior, University of Minnesota, 1479 Gortner Avenue, St. Paul, MN 55108 USA; 2 Department of Plant Pathology, University of Florida, 2550 Hull Road, Gainesville, FL 32611 USA; 3 Department of Plant and Microbial Biology, University of Minnesota, 1479 Gortner Avenue, St. Paul, MN 55108 USA; 4 Department of Biological Sciences, Smith College, 44 College Lane, Northampton, MA 01063 USA

**Keywords:** Mycorrhizal mutualisms, Biotic interactions, Seedling establishment, Assisted migration, Plant-soil feedbacks

## Abstract

Rapid climate change imperils many small-ranged endemic species as the climate envelopes of their native ranges shift poleward. In addition to abiotic changes, biotic interactions are expected to play a critical role in plant species’ responses. Below-ground interactions are of particular interest given increasing evidence of microbial effects on plant performance and the prevalence of mycorrhizal mutualisms. We used greenhouse mesocosm experiments to investigate how natural northward migration/assisted colonization of *Rhododendron catawbiense*, a small-ranged endemic eastern U.S. shrub, might be influenced by novel below-ground biotic interactions from soils north of its native range, particularly with ericoid mycorrhizal fungi (ERM). We compared germination, leaf size, survival, and ERM colonization rates of endemic *R. catawbiense* and widespread *R. maximum* when sown on different soil inoculum treatments: a sterilized control; a non-ERM biotic control; ERM communities from northern *R. maximum* populations; and ERM communities collected from the native range of *R. catawbiense*. Germination rates for both species when inoculated with congeners' novel soils were significantly higher than when inoculated with conspecific soils, or non-mycorrhizal controls. Mortality rates were unaffected by treatment, suggesting that the unexpected reciprocal effect of each species’ increased establishment in association with heterospecific ERM could have lasting demographic effects. Our results suggest that seedling establishment of *R. catawbiense* in northern regions outside its native range could be facilitated by the presence of extant congeners like *R. maximum* and their associated soil microbiota. These findings have direct relevance to the potential for successful poleward migration or future assisted colonization efforts.

**Supplementary Information:**

The online version contains supplementary material available at 10.1007/s00442-021-05081-9.

## Introduction

In the face of rapid anthropogenic climate change, large numbers of species will need to shift their geographic distributions poleward to track the climatic conditions that have supported their populations in the past (Chen et al. 2011; Corlett and Westcott 2013; Diffenbaugh 2013). It is feared that the migration rates necessary to keep pace with these changes will exceed many species’ capacities for dispersal and colonization, risking widespread extinctions (Thomas et al. 2004). A further complication and concern on this topic is that many studies forecasting range shifts use approaches such as species distribution models, which focus almost exclusively on abiotic factors, like temperature, precipitation, and soils (Elith and Leathwick 2009; Franklin and Miller 2010). However, ecological and evolutionary theory (Hochberg and Ives 1999; Case and Taper 2000; Gravel et al. 2011), and a growing body of empirical studies (Moeller et al. 2012; Afkhami et al. 2014; Baer and Maron 2018; Benning et al. 2019; Benning and Moeller 2019), suggest that biotic interactions are also key to understanding many species’ current distributions, abundances, and range limits (reviewed in Louthan et al. 2015). These biotic interactions might strongly influence extinction risk and the capacity for natural range shifts in the context of climate change (Hille Ris Lambers et al. 2013), and would also be crucial to consider in the context of “assisted migration” or “assisted colonization” conservation efforts (McLachlan et al. 2007; Brodie et al. 2021).

In the context of biotic limits on migration and range shifts, species dependent on obligate mutualisms, or those with facultative mutualisms important to seedling establishment, are of particular concern (Dunn et al. 2009). For such species, it is possible that a lack of required mutualists in a new region, or the presence of poorly-matched novel partners, could hinder the range shifts necessary to track rapid changes in the geographic distribution of abiotically-suitable habitat (Parker 2001; van der Putten 2012). At an extreme, these dynamics could also lead to geographical decoupling of co-dependent mutualistic partners, for example, if the species involved responded differently to changing abiotic conditions (van der Putten 2012; Lankau 2016) or if they differed in dispersal and migration ability, risking accelerated decline or even coextinction.

Although the possibility that a requirement for positive biotic interactions (e.g., mutualism or facilitation) might hinder migration and colonization capacity has received some conceptual attention, limited empirical research has been conducted to date. In contrast, the role of antagonistic biotic interactions, or the lack thereof, has received considerable attention, especially in the context of “enemy escape” and the emergence of invasive, exotic species released from antagonistic biotic pressures in new regions (Keane and Crawley 2002; Mitchell and Power 2003). However, increasing numbers of studies have highlighted the role of positive interactions, or lack thereof, on species’ current distributions and their potential to establish in new regions (Moeller et al. 2012; Afkhami et al. 2014; Osborne et al. 2018; Moyano et al. 2020). In particular, evidence is emerging that positive below-ground interactions might impact colonization capacity (Parker et al. 2006; Nuñez et al. 2009). For example, Delavaux et al. (2019) showed that plant species with mycorrhizal associations are less common on islands, where their fungal symbionts are likely absent, than in mainland floras. In contrast, other studies have recently highlighted the potential for novel mutualistic partnerships (e.g., mycorrhizae) to enhance naturalization of non-native plant species (Moyano et al. 2020). These studies suggest that biotic interactions could play an important, but sometimes unpredictable, role in facilitating or hindering plant colonization and migration at large biogeographical scales.

The relevance of mutualistic interactions to understanding plant migration potential likely varies greatly across species. In extreme cases, plant species involved in obligate mutualisms may be locked into precarious dynamics in which the presence of a key partner is critical for establishment and spread in new regions. Exotic plant invasions provide some striking examples of this possibility: for example, several species of fig trees (*Ficus* spp.) planted beyond their native ranges were sterile during their initial horticultural introductions, but became reproductive (and invasive) only after their obligate pollinator wasps were also introduced (Nadel et al. 1992; Gardner and Early 1996). By contrast, species involved in facultative mutualisms with broadly-distributed partners (e.g., generalist pollinators or mycorrhizal fungi) or with traits reducing requirements for mutualism (e.g., self-pollination: Grossenbacher et al. 2015) might not face such a significant “mutualism filter” on migration and colonization ability (Policelli et al. 2018).

Even for those species that show some level of dependency on specialized mutualisms, determining its effect on migration potential is likely to be complex, idiosyncratic, and often context-dependent. In the case of taxa from species-rich clades, individual taxa may share, or at least be compatible with, their congeners’ partners (e.g., as seen among some European *Orchis* spp. with specialized mycorrhizae; Jacquemyn et al. 2011), raising the possibility that potential partners might be available where congeners already occur in new regions, even if the target species has not yet colonized the region. However, adding to this complexity is the likelihood that many intimate or symbiotic mutualisms undergo localized coevolution, such that the benefits received from a common partner may shift from one population, species, or region to another (Thompson and Cunningham 2002; Hoeksema 2010; Johnson et al. 2010). In this context, even migration into new regions where potential partners are already present raises the possibility that locally- and reciprocally-adapted mutualisms will be lost and unpredictable new partnerships formed. These novel interactions among “naïve” partners could lead to a wide range of possible outcomes, from mutualism to parasitism (Lankau and Keymer 2018). These unpredictable dynamics could theoretically result in a range of consequences for plant colonization, with possible effects ranging from biotic resistance at one extreme to increased establishment through facilitation at the other. Clearly, gaining a better understanding of the possible outcomes when mutualisms are lost, disrupted, or altered during natural migration or with planned assisted colonization efforts will be crucial for ensuring the survival of many climate-threatened species in the future.

### Case study: *Rhododendron* and ericoid mycorrhizal fungi

Among plant taxa with specialized below-ground mutualisms, the Ericaceae stand out as a clade that is both diverse and highly consequential for ecosystem processes in many temperate and boreal regions (Cairney and Meharg 2003). Members of the Ericaceae, including shrubs in the genus *Rhododendron*, form specialized relationships with ericoid mycorrhizal fungi (ERM) that are distinct from the more generalist arbuscular mycorrhizal fungi (AM) and ectomycorrhizal fungi (EM) seen in many other plant lineages (Read 1996). Fungi involved in ERM symbioses penetrate the epidermal cells of ericaceous plant’s fine “hair roots” and form coiled hyphal complexes where nutrient and resource transfers between plant and fungi take place (Perotto et al. 2012). The fungi are capable of decomposing organic material in the soil to access N, a valuable trait for the fungi and host plant in habitats that are otherwise often nutrient-poor and stressful (Kerley and Read 1998; Cairney and Meharg 2003; Lin et al. 2011). This specialized mutualism is apparently critical for the Ericaceae clade’s ability to thrive in, and often dominate, plant communities on acidic, nutrient-poor, or heavy metal-saturated soils (Cairney and Meharg 2003).

In addition to their role in the survival and performance of adult Ericaceae, there is also evidence of critical mutualistic and symbiotic interactions at earlier plant life stages. Many members of the Ericaceae produce relatively small “dust” seeds that lack substantial resources for growth and establishment beyond the seed germination stage, and given the family’s common association with nutrient-poor habitats, early interactions with ERM could be critical to seedling survival and growth. For example, Wei et al. (2016) found that the nitrogen content and biomass of *Rhododendron fortunei* seedlings was markedly greater in experimental ERM- colonized plants compared to control plants. In the field, such differences could easily be decisive in determining the success or failure of individual seedling establishment, as well as population growth rates.

The nature and exclusivity of the symbiotic mutualism seen between Ericaceae and ERM fungi is not entirely clear. Individual ERM fungi may associate with multiple plant species or individuals through interconnected hyphal networks (van der Heijden et al. 2015). Similarly in Ericaceae roots, including *Rhododendron*, multiple occupancy of various ERM species and genotypes has been documented (Perotto et al. 2012; Tian et al. 2010)*.* This diversity of ERM fungi might reflect the potential for Ericaceae to partner adaptively with a range of ERM, thus expanding the functional diversity of the mutualism. It may also be that host plants experience differential fitness costs and benefits to sustaining mutualisms with distinct but functionally overlapping or redundant partners (Klironomos 2003).

The extent to which these important partnerships could be maintained or lost during long-distance migration or colonization of a new region is unclear. However, ERM are typically observed as sterile, asexual fungi, with few observations reported of spore-bearing apothecia (Smith and Read 2008); as such, the prospect of both seeds and spores from a locally co-occurring ericaceous plant × ERM mutualism dispersing long distance and jointly colonizing a remote new location appears vanishingly small.

**Fig. 1:**
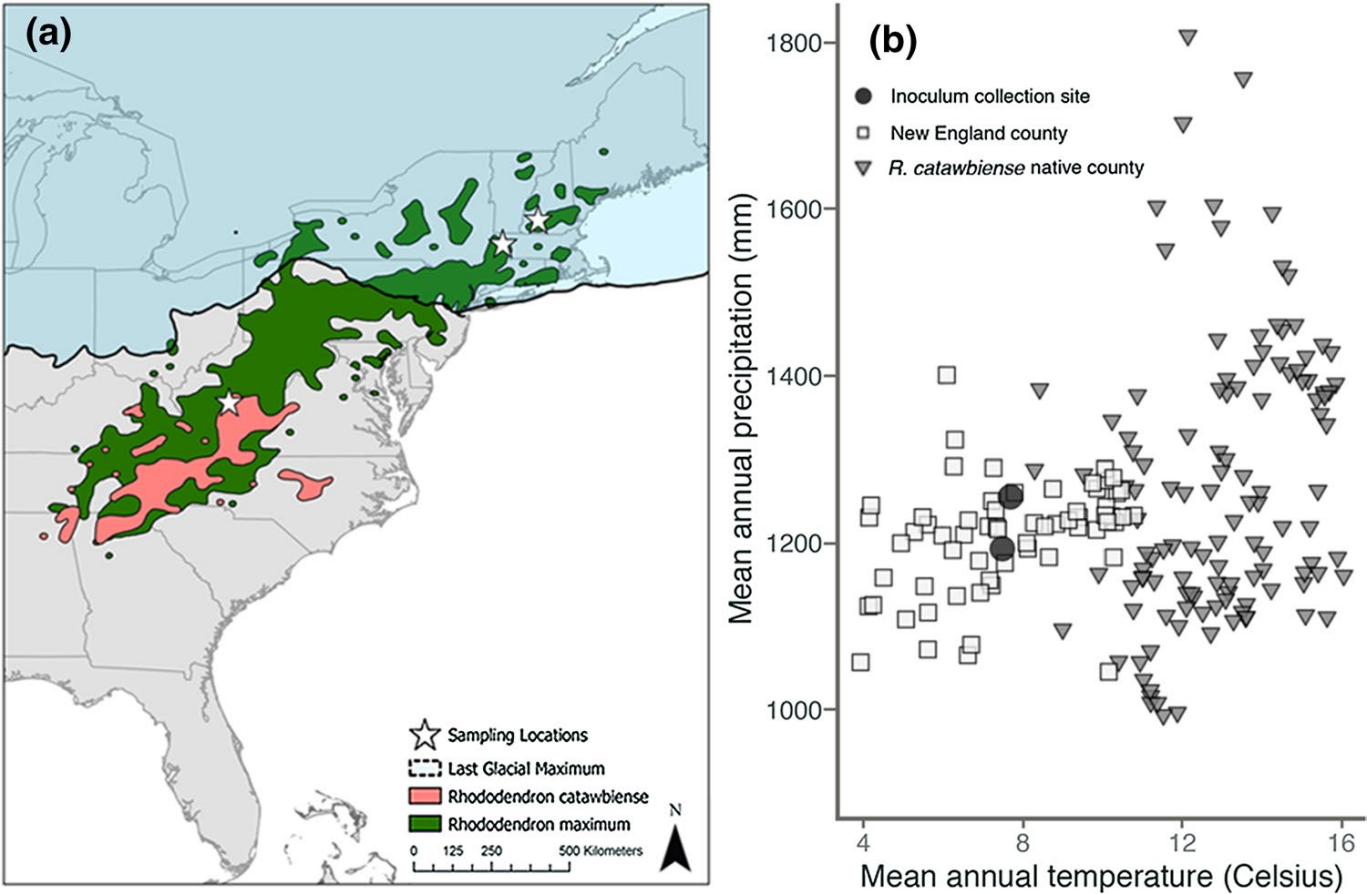
**a** Map of the current ranges of *R. catawbiense* (pink) and *R. maximum* (green) in relation to the extent of the Last Glacial Maximum (circa 20,000 years ago). Stars indicate the locations of soil inoculum and seed collection for Experiments 1 and 2. **b** Macroclimate features of the native range of *R. catawbiense* and the New England region where this work was conducted. Conditions at inoculum collection sites are indicated. Data points correspond to county-level averages of 1981–2010 climate normals for relevant counties in Connecticut, Maine, Massachusetts, New Hampshire, Rhode Island, and Vermont (PRISM Climate Group, Oregon State University, http://prism.oregonstate.edu)

To begin to understand how these important mutualistic interactions might influence the potential for *Rhododendron* species to establish in new regions beyond their native range, we conducted an experiment focusing on two eastern U.S. native *Rhododendron* species: *R. maximum* L. and *R. catawbiense* Michx. These species differ substantially in range size: great laurel rhododendron (*R. maximum*) is distributed from the southern Appalachian Mountains northward into New England, including areas several 100 km to the north of the Last Glacial Maximum of the Pleistocene epoch, indicating successful long-distance migration in the past (Fig. 1a). By contrast, Catawba rhododendron (*R. catawbiense*) is endemic to a more limited region, occurring at high elevations in the central and southern Appalachian Mountains, several 100 km south of the Last Glacial Maximum (Fig. 1a) (Kartesz 2014).

The distribution pattern of *R. catawbiense* is similar to that of many other range-restricted forest plant species that are associated with the Southern Appalachian Mountains biodiversity hotspot in the eastern U.S., and it is suspected that the distributions of these endemic species might trace in part to limited dispersal and migration from Pleistocene refugia in the south (Bellemare and Moeller 2014). In the face of modern climate change, small-ranged species from the southern Appalachians Mountains might be at high risk and could become candidates for poleward assisted colonization into the northeastern U.S., a region with similar forest habitats and cooler climate (Bellemare and Moeller 2014; Bellemare et al. 2017; Fig. 1b*).* However, the role that biotic interactions, especially with mutualistic ERM, might play in natural or human-assisted long-distance colonization could be decisive and is not well understood.

Our primary objectives in this study were to: (1) investigate the influence of whole-soil ERM inoculation on *Rhododendron* seed germination, seedling growth, and mortality, (2) to explore the ecological effects of *Rhododendron* interaction with local versus novel ERM communities for two species: the endemic *R. catawbiense* and the more widespread *R. maximum,* and (3) for the endemic *R. catawbiense*, to assess whether interactions with ERM communities from outside its range to the north might facilitate or hinder potential northward colonization.

## Materials and methods

### Experiment 1: Effect of sterilized soil vs live inoculum on *Rhododendron* seed germination and growth

Our first experiment aimed to determine if *R. catawbiense* plants could form effective associations with novel northern ERM from outside the species’ native range, and if so, reveal how their germination and growth rates were affected compared to seeds without ERM. This experiment also compared outcomes of novel *R. catawbiense*/northern ERM pairings compared to local pairings of *R. maximum*/northern ERM. In Fall 2014, seeds from *R. catawbiense* were collected from a horticultural specimen (1062PA*) grown in the Smith College Botanic Garden, Northampton, MA, and seeds from *R. maximum* were collected from a wild population in Fitzwilliam, NH.

To capture representative samples of ERM communities in the north, soil organic layer material was collected from two locations with *R. maximum* in New England: the *R. maximum* seed source population in Fitzwilliam, NH and a second site in Whately, MA with *R. maximum* and another native congener, *R. periclymenoides* (Michx.) Shinners. The bulk organic material samples from the two sites, including *Rhododendron* fine roots, were pooled and homogenized, and subsampled to inoculate the treatment mesocosms described below.

Sixty replicate mesocosms were established in December 2014 using 946 mL plastic containers filled with a 50:50 sand and peat moss mix; drainage holes were drilled into the bottom of each container and the central 6 × 6 cm of each lid was removed to enhance light and air flow. To this base mix of soil, ~ 60 mL of the homogenized wild northern soil inoculum was added to the soil surface. The mesocosms were watered to capacity, allowed to drain, and then half were microwaved until the soil temperature reached ~ 100 °C to sterilize the soil and eliminate soil biota (Trevors 1996). All mesocosms (live inoculum and sterilized) were placed in a greenhouse (Lyman Plant House at the Smith College Botanic Garden) for 6 weeks with temperatures in the range of 8–15 °C and natural December–January day lengths (~ 9 h light). In late January 2015, half the mesocosms (15 sterilized and 15 live-inoculated) were each sown with 50 *R. catawbiense* seeds, and the other half were sown with 50 *R. maximum* seeds each, by scattering seeds on the soil surface in the central portion of each mesocosm container. The containers were then placed in an array on greenhouse benches with temperatures between 23–26** °C** and a 12 h light/dark cycle maintained via natural and supplemental light.

Starting in February 2015, the mesocosms were monitored daily for evidence of seed germination. Total germination rate was tallied based on the maximum number of seedlings observed per mesocosm during this time. Established seedlings were measured for leaf size in April 2015 (~ 3 months after initial seed germination), with the length of largest leaf of the 5 largest seedlings in each mesocosm being recorded.

In Spring 2017, we conducted a separate experiment as a modified version of the first experiment, replacing the sterilized control treatment with a non-mycorrhizal biotic control, to assess how germination patterns might be affected by inoculum collected from different sites and with a live, but non-ERM, microbial community in the control treatment (Online Resource 1). This was intended to reveal whether our prior findings would be repeated, setting the stage for us to conduct Experiment 2.

### Experiment 2: Effect of four inocula on *Rhododendron* seed germination and plant growth

#### Mesocosm establishment

We conducted Experiment 2 in Spring 2018 to corroborate our previous findings with additional measurements and treatments. We investigated whether *R. catawbiense’s* partnership with novel ERM resulted in different seed germination and seedling performance outcomes than when partnered with its own local ERM, as well as with live whole-soil communities that did not contain ERM, and compared these results to the performance of *R. maximum* in the same treatments. In addition to the original treatment with *R. maximum* inoculum from Experiment 1, the second iteration of the experiment included wild-collected seed and soil inoculum from native populations of *R. catawbiense* near Medo, WV in October 2017, with seed capsules collected from ten different *R. catawbiense* individuals and soil inoculum with fine roots collected from beneath the same ten plants (Fig. 1a). The soil inoculum included mostly organic horizon material, but also mineral soil (A horizon) from under two plants that were growing directly in mineral soil. For *R. maximum*, seeds and organic horizon soil inoculum were collected from below 10 individuals in a wild population in Shelburne, MA, following the same protocols (Fig. 1). Seeds were pooled for each species, homogenized, and then randomly subsampled. Similarly, each set of soil inoculum samples was pooled by species, homogenized, and refrigerated at 3–4 °C until experimental setup.

In addition to the targeted *Rhododendron* soil inoculum described above, a second set of soil organic layer material was collected in November 2017 from a conifer-dominated forest site lacking Ericaceae at Smith College’s MacLeish Field Station in Whately, MA. Due to the absence of Ericaceae plants in the area sampled, this organic layer material was presumed to lack high abundances of ERM, but likely still contained a diverse assemblage of typical non-ERM forest soil fungi and microbes. We elected to use this organic layer material (hereafter “forest soil”) as part of the base soil mixture for the mesocosms, as it was expected to include a functioning forest soil microbial community, unlike a sterilized control, allowing us to more directly test the unique effects of the presence vs. absence of ERM, not confounded by an underlying lack of normal soil microbes in a fully sterilized soil. The forest soil was refrigerated at 3–4 °C until late February 2017, then homogenized and combined with washed, coarse sand (Quikcrete All-Purpose Sand no. 1152) in a 6:5 ratio to form the base potting material for the experiment.

The containers used for mesocosms in Experiment 2 were slightly larger (1182 mL; ziplock) but otherwise similar to the prior experiments. The bulk soil mix was placed into 150 mesocosms, where 50 of these then received ~ 80 mL of homogenized *R. catawbiense* soil organic layer inoculum, 50 received the same amount of the *R. maximum* inoculum, and 50 received an additional ~ 80 mL supplement of the non-*Rhododendron* forest soil. The latter treatment ensured that the soil in the third treatment had a similarly organic material-rich surface to that of the other two treatments. Each mesocosm was watered to capacity, allowed to drain, and placed in the greenhouse in late February 2018 under the same conditions as the first experiment.

After 1 week, half of the mesocosms within each soil treatment were sown with 20 *R. catawbiense* seeds each, and the other half sown with 20 *R. maximum* seeds each, maintained using the same methods as the initial experiment. Mesocosms for the two species and three treatments were systematically alternated along greenhouse benches and maintained as in Experiment 1 with 12-h light:dark cycle.

#### Germination and growth

Approximately 3 weeks after seed sowing, weekly surveys were initiated to score germination and continued for 3 weeks. The final week’s germination count was the set of observations used in statistical analysis of germination rates; these numbers reflected the highest seedling counts in the containers, after which no more seedlings germinated. It was not feasible for us to track the survival and mortality of individual seedlings without disturbing the mesocosms significantly due to the seedlings’ small size and proximity, so we quantified the overall germination and mortality rates in each mesocosm. Seedling survival and growth was then monitored for an additional 3 months during the spring and early summer of 2018. Performance data collected during this time included the length of the largest leaf on the five largest seedlings in each mesocosm (measured with digital calipers) at two time points (May 17th–19th and again 3 weeks later from June 7th–9th). We chose to measure only the five largest seedlings because while many seedlings germinated, only a fraction began to develop into juveniles with true leaves over the course of the experiment. Finally, on June 11th, mortality rate was estimated as the difference between the total number of germinants detected in the final April germination survey and the number of seedlings still alive on June 11th.

#### Evaluating ERM colonization using light microscopy

We used light microscopy to evaluate if plant performance outcomes between species and treatments could be attributed to differences in amount of ERM root colonization alone, as well as to assess the efficacy of our live control and ERM soils (i.e. the assumption that the control plants would have very low colonization rates). Whole-root samples from three seedlings per mesocosm, selected randomly from the largest five seedlings of each mesocosm, were harvested on June 13th 2018, and stained for ERM detection with light microscopy. To do this, seedling roots were washed, combined in a stainless steel tea strainer, cleared in boiling (100 °C) 10% KOH (wt/vol) solution for five minutes, rinsed in deionized water, and then stained in boiling 5% black Sheaffer ink and vinegar solution (5% acetic acid) for five minutes. The stained roots were then rinsed in 500 mL deionized water acidified with 1 ml of acetic acid (Vierheilig et al. 1998) and stored in deionized water.

To assess ERM colonization of seedlings, the stained roots were imaged using 20–40 × magnification on an Olympus CKX41 inverted light microscope with Excelis digital camera to detect the presence of the distinctive, darkly-stained ERM hyphal structures produced inside root epidermal cells. Four random root tips were selected from each plant, and level of colonization within a given field of view (1.5 mm diameter) was visually categorized as: 0% colonization, between 1–25% of root length colonized, 26–50%, 51–75%, or 76–100% colonized. Each root tip was then assigned a value equal to the midpoint of its associated bin.

#### Evaluating ERM colonization using scanning electron microscopy

In May 2018, a whole-root sample from one *Rhododendron* seedling per mesocosm (again selected randomly from among the largest 5 seedlings of each mesocosm) was harvested for scanning electron microscopy (SEM). We used SEM to better elucidate whether fungal hyphae observed with light microscopy exhibited characteristics consistent with ERM, such as colonization of only the root epidermal cells with dense hyphal coils, unlike arbuscular mycorrhizal fungi. Washed root samples were fixed overnight with 2.5% glutaraldehyde in 0.1 M cacodylate buffer, rinsed in cacodylate buffer, subjected to two hours of post-fixation in 1% osmium tetroxide, rinsed in deionized water, and dehydrated in a progression of EtOH concentrations up to 100%. The dehydrated root samples were then frozen with liquid nitrogen and cross-sectioned, sputter coated with palladium gold, and imaged in an FEI Quanta 450 Scanning Electron Microscope.

In addition to observing established seedling roots from Experiment 2 with SEM, this technique was also used on a third, non-experimental set of *R. catawbiense* seeds to document whether physical contacts between germinating seeds and fungal hyphae were present at the earliest stages of germination. For this work, a second set of *R. catawbiense* seeds were sown into eight additional mesocosms established in May 2018 with *R. maximum* inoculum. After 20 days, visibly germinating seeds were removed from the mesocosms, rinsed, fixed, coated, and imaged as described above for whole-root samples.

### Data analyses

For Experiment 1, we tested for the effects of two soil treatments (*R. maximum* inoculated vs. sterilized control), plant species identity (*R. maximum* vs. *R. catawbiense*), and their interaction on seed germination using a likelihood ratio test on a generalized linear binomial model with a logit link. We used generalized mixed-effect linear models with *F*-tests to examine whether treatment, species, and the interaction resulted in differences in largest leaf size among treatments and species, using the log-transformed mean of five leaf measurements within each mesocosm, including mesocosm as a random effect.

For Experiment 2, we tested for the effects of three soil inocula (*R. catawbiense* soil inoculum, *R. maximum* soil inoculum, and control forest soil), plant species identity (*R. maximum* vs. *R. catawbiense*), and their interaction on seed germination and seedling mortality using generalized linear binomial models with logit links and likelihood ratio tests. We fitted a generalized mixed-effect linear model to test whether inocula, species, and the interaction resulted in differences in largest leaf size among treatments and species. We used the log-transformed values of each individual’s largest leaf size to mitigate heteroskedasticity of residuals, and included mesocosm as a random effect, and performed a likelihood ratio test.

Since the root colonization measurements were assigned as bin midpoints rather than exact measurements, we averaged them over each individual to provide a more meaningful assessment of the colonization level of a given plant. After staining, while we retained treatment and species information, we lacked data on which mesocosm each of the three harvested individuals were associated with, and thus could not include it as a random effect. As such, we chose to instead fit a linear model to test whether inocula, species, and the interaction resulted in differences in percent root colonization by ERM among treatments and species. For these analyses, we log-transformed the means of each individual’s root colonization levels to meet assumptions of residual homoskedasticity and performed *F*-tests.

For all analyses, we tested for significant differences in nine pre-planned contrasts (within both species across treatments, and within all three treatments across species) using Tukey’s tests with a Bonferroni-Holm correction applied.

We conducted binomial models using the [glm] package, generalized linear models using the [nlme] package, *F*-tests and likelihood ratio tests using the [car] package, and pairwise tests using the [emmeans] package in R (Version 3.1.5, 2018).

## Results

### Experiment 1: Effect of two soil inocula on *Rhododendron* seed germination and growth

#### Germination

**Fig. 2:**
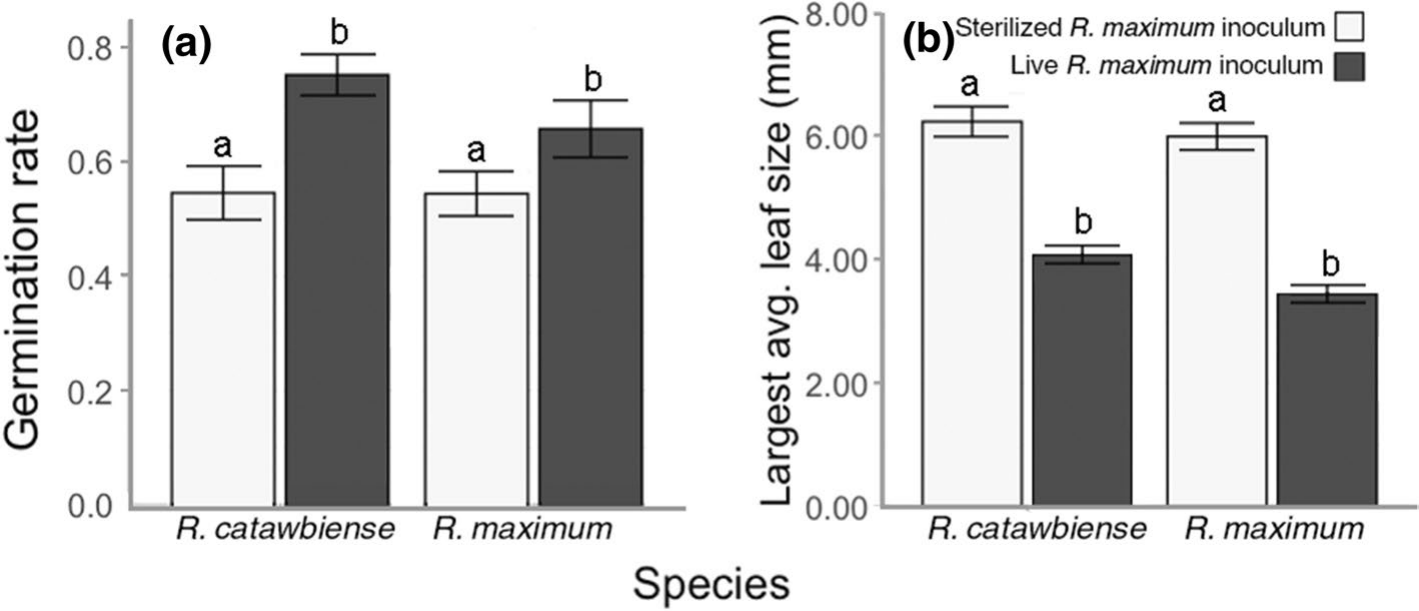
Inoculum treatments effects on **a** germination rate and **b** largest leaf size of *R. maximum* and *R. catawbiense* in Experiment 1. Largest leaf size was measured 3 months after seed sowing. Values are means ± SE; *N* = 15 (**a**)/75 (**b**). Significant Tukey's test differences with Bonferroni-Holm correction (*P* < 0.05) among categories within each sub-panel are indicated with differing letters

The interaction of inoculum treatment and species identity (*P* < 0.003) was a highly significant predictor of germination rate (Fig. 2a). Germination rate of *R. catawbiense* seed was significantly higher in mesocosms with the novel *R. maximum* inoculum (75.2% ± 3.6% SE) than in mesocosms with the sterilized control (54.5% ± 4.7% SE, *P* < 0.001) (Fig. 2a). Germination rate for *R. maximum* seed was also significantly higher in the inoculum treatment (65.7% ± 4.99% SE) compared to the sterilized control (54.4% ± 3.9% SE, *P* < 0.001). The *R. catawbiense* germination rate was significantly higher than *R. maximum* germination in the live treatment (*P* < 0.001); however, there was no difference in germination rate between species in the sterilized control (*P* = 0.950) (Fig. 2a).

#### Leaf size

Average largest leaf size (hereafter LLS) was influenced significantly by soil treatment (df = 1, *χ*^2^ = 27.674, *P* < 0.001). *R. catawbiense* grew significantly larger leaves in the sterilized control (6.24 mm ± 0.244 SE) than in the live inoculum treatment (4.08 mm ± 0.145 SE, *P* = 0.006) (Fig. 2b). *R. maximum* followed a similar pattern of larger leaves in the control (6.00 mm ± 0.218 SE) than the live treatment (3.45 mm ± 0.142 SE, *P* < 0.001). There was no difference in LLS between species in the sterilized control (*P* = 0.693) or in the live inoculum treatment (*P* = 0.315) (Fig. 2b).

### Experiment 2: Effect of three inocula on *Rhododendron* seed germination, mortality, growth, and root mycorrhizal colonization

#### Germination

**Fig. 3:**
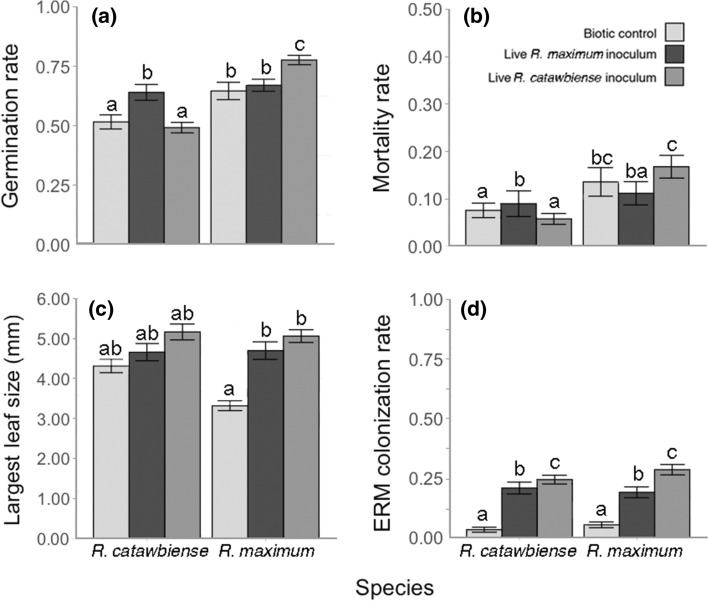
Mean responses (± SE) of *R. maximum* and *R. catawbiense* to two soil inoculation treatments and biotic controls in Experiment 2. **a** Germination rates of the two species when seeds were exposed to soils inoculated with organic material from conspecific versus heterospecific seed source sites and control. **b** Mortality rates, **c** mean largest leaf size and **d** mean rates of ericoid mycorrhizal fungi (ERM) colonization on roots of the two species quantified 3 months after initial seed sowing in 2018 on soils inoculated with conspecific and heterospecific organic material, and control. Significant Tukey's test differences with Bonferroni-Holm correction (*P* < 0.05) among categories within each sub-panel are indicated with differing letters

Germination rate was significantly affected by the interaction of soil inoculum treatment and species identity (df = 2, *χ*^2^ = 36.4, *P* < 0.001). Both *Rhododendron* species’ germination rates were significantly higher when planted in novel heterospecific inoculum. However, for both species, there was no significant difference between control mesocosms and those inoculated with the species’ own local soil (Fig. 3). The germination rate for *R. catawbiense* with novel *R. maximum* inoculum (64.0% ± 3.33% SE) was significantly higher compared to mesocosms with control inoculum (51.6% ± 2.97% SE, *P* < 0.001) or its own local inoculum (49.2% ± 2.19% SE, *P* < 0.001). Germination rate for *R. maximum* was also significantly higher in mesocosms with the novel *R. catawbiense* inoculum (77.6% ± 1.96% SE) compared with the control inoculum (64.6% ± 3.65% SE, *P* < 0.001) and its own local northern inoculum (67% ± 2.53% SE, *P* = 0.011).

#### Largest leaf size

LLS was influenced significantly by soil inoculum (df = 2, *χ*^2^ = 18.7, *P* < 0.001). Both species followed a similar pattern, with each displaying the smallest leaves in the control, intermediately sized leaves in the *R. maximum* inoculum, and the largest leaves in the *R. catawbiense* inoculum, with some significant differences in magnitude (Fig. 3c).

#### Seedling mortality

Species identity and inoculum treatment interacted to produce a significant effect on seedling mortality (df = 2, *χ*^2^ = 9.61, *P* = 0.008). There were no significant pairwise differences in mortality rate between soil inoculum treatments for either species (Fig. 3b). Within soil treatments, *R. catawbiense* seedlings died at a slightly lower rate (7.6% ± 1.53% SE) than *R. maximum* in the control (13.6% ± 3.01 SE, *P* = 0.018), and at a much lower rate (5.8% ± 1.14% SE) than *R. maximum* seedlings (16.8% ± 2.39% SE) in the *R. catawbiense* inoculum treatment (*P* < 0.001). No differences in species mortality were observed within the *R. maximum* inoculum treatment (Fig. 3b).

#### Mycorrhizal colonization rate

In the analysis of ERM seedling root colonization rates, the only significant model term was soil treatment (df = 2, *F* = 145.4, *P* < 0.001). Both species displayed a similar parallel pattern across treatments, with very low colonization rates in the control, intermediate colonization rates in *R. maximum* soil, and the highest rates in *R. catawbiense* soil (Fig. 3d). Within treatments, no differences in ERM colonization rates between the two species were observed.

#### Scanning electron microscopy

**Fig. 4:**
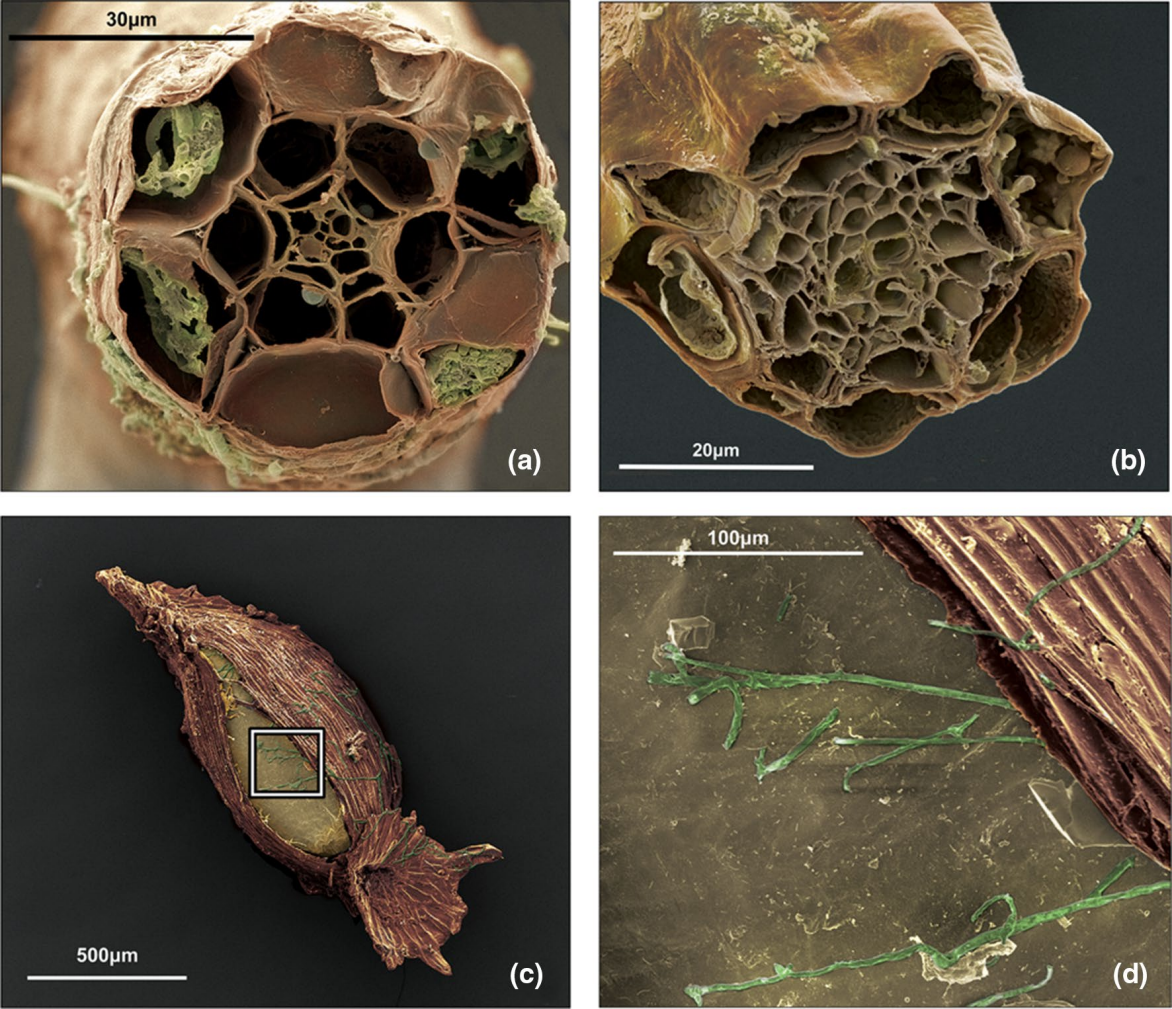
**a** Colorized SEM image of an *R. catawbiense* root cross-section with ericoid hyphal coils visible inside the epidermal cells, from a mesocosm inoculated with *R. maximum* (novel) soil, with roots colored brown and fungal hyphae colored green. **b** Colorized SEM image of an *R. catawbiense* root cross section with no ERM in the root, from a mesocosm inoculated with *R. maximum* soil. **c** Germinating *R. catawbiense* seed with fungal hyphae in contact with the germinating seed. **d** Closeup of fungal hyphae contacting interior tissues of seed as seed coat splits during germination

Our SEM images of cross-sectioned *R. catawbiense* and *R. maximum* roots from the seedlings planted in both types of mycorrhizal inoculum revealed the presence of ERM hyphal complexes in the roots, so identified by their characteristic hyphal coils within only epidermal cells (Fig. 4a–b). Additionally, our SEM images of germinating seeds revealed the presence and interaction of fungal hyphae with emerging plant radicles at the first stages of germination (Fig. 4c–d).

## Discussion

The results of these experiments provide evidence that seeds of both *R. catawbiense* and *R. maximum* exhibit significantly higher germination rates on soils containing novel soil biota and ERM communities than on soils containing their own local biota and ERM, or on non-ERM biotic controls or sterilized controls. This unexpected pattern was observed for the endemic *R. catawbiense* seeds in both germination experiments reported here, as well as in our second trial of Experiment 1 with different inoculum sources (Online Resource 1), confirming its reproducibility and consistency. These findings have striking implications for gauging the ability of an endemic species like *R. catawbiense* to shift its distribution poleward in response to climate change. Rather than experiencing a performance-reducing decoupling from its locally co-adapted ERM partners inside its native range, our results suggest that this endemic species might encounter novel ERM partners in the north that could maintain, or even enhance, the ability of its seeds to germinate and establish in new regions, as long as more widespread congeners (e.g., *R. maximum*) are already present in the area and hosting ERM fungi. Further, because population dynamics of long-lived *Rhododendron* appear to be highly dependent on the sensitive seedling stage, even modest increases in germination and establishment rates, as detected in this study, could have substantial effects on long-term population growth (e.g., Erfmeier and Bruelheide 2004).

In Experiment 1, both *R. maximum* and *R. catawbiense* germinated at higher rates in the live-inoculated soil than in the sterilized control (Fig. 2a). However, the pairing of *R. catawbiense* with novel ERM and soil biota in the live inoculum from *R. maximum* sites had a significantly higher germination rate than the pairing of *R. maximum* with ERM and soil biota from its home site and region. The similar germination rates of these two species in the sterilized treatment suggests that there is some unique, positive benefit from pairing of novel partners (i.e., *R. catawbiense* seeds + *R. maximum* soil inoculum) that cannot be attributed to underlying differences between the species in seed viability or other innate factors. This pattern of increased germination on novel soil inocula from heterospecific sources was also detected in the repeated trial of Experiment 1 (Online Resource 1), and most strikingly for both study species in Experiment 2. Notably, this effect seems to have the potential for persistent effects on later demographic dynamics, as it was not offset by subsequent seedling mortality, which remained generally consistent among treatments and species.

There are some important caveats to these results. First, it was notable in Experiment 1 that while both species germinated at higher rates in the ERM inoculated mesocosms, leaf growth was highest in the sterilized control. This pattern could trace to several possibilities: for example, without an ERM partner, *Rhododendron* seedlings might have been able to direct more photosynthates to their own growth; alternatively, the sterilized soils may have yielded an environment where seedlings did not have to compete with microbes or fungi for soil nutrients, resulting in a shift to more investment in above-ground growth. We can also not rule out the possibility of “enemy release” in the sterilized soil, if soil pathogens associated with the inoculum from adult *R. maximum* negatively impact leaf growth rates in *Rhododendron* seedlings (but not seed germination). The relevance of these particular findings from the sterilized soil to plant performance in the wild, where diverse soil microbiota are ubiquitous, and ERM might be crucial to obtaining scarce soil nutrients, is unclear. For this reason, we believe the live biotic control used in Experiment 2 is a more relevant benchmark for assessing performance and the effects of different inoculum types.

Second, in Experiment 2, increased seed germination when exposed to novel inoculum types did not fully translate to increased growth in later life stages. R*. maximum* showed higher leaf growth in mesocosms inoculated with southern ERM than in control mesocosms. *R. catawbiense,* however, did not exhibit any difference in growth between treatments. Both species were colonized by ERM at the highest rates in *R. catawbiense* inoculum soil, followed by *R. maximum* inoculum soil, with minimal colonization in the biotic control. This suggests that the quality or mutualistic benefit of ERM for later life stages of *Rhododendron* could also differ between sites or regions, possibly as a result of differing levels of root colonization.

### The complex and dynamic nature of mutualisms

Previous studies have noted germination-growth tradeoffs in ERM mutualisms in vitro (Grelet et al. 2009; Jansa and Vosátka 2000), highlighting the context-dependency of mutualisms across lifespan in addition to geographic space. Tradeoffs inherent to mycorrhizal partnerships between plant, soil origin, and fungus have been thought to explain differences in growth rates between locally co-adapted and novel partnerships (Rúa et al. 2016). For example, Johnson et al. (2010) found that locally adapted partnerships between grasses and their communities of AM conferred more mutualistic benefits to the plants than novel partnerships. Earlier experiments by Johnson et al. (1997) also showed high levels of local co-adaptation between plants and their mycorrhizal fungi. Our results for both *Rhododendron* seed germination and leaf growth generally stand in contrast to the expectation drawn from these studies that locally co-adapted partnerships will outperform novel partnerships between plants and their mutualistic partners. First, we repeatedly found a significant pattern of seeds germinating at higher rates when interacting with novel ERM communities collected from below heterospecific *Rhododendron*; indeed, for Experiment 2, each species’ local ERM inoculum did not differ significantly from neutral, non-ERM forest soil controls. It is notable that previous research with another member of the Ericaceae (*Monotropa uniflora*) detected increased seedling development when plants partnered with novel *Russula* spp. fungi differing from that of plants in their source site (Bidartondo and Bruns 2005). Second, in Experiment 2, at the seedling life stage, we detected signs of a regional effect in which both *Rhododendron* species showed higher rates of ERM colonization in their roots when exposed to live inoculum from the more southern population of *R. catawbiense*. Thus, it seems that novel ERM fungal partners may increase *Rhododendron* germination rates, but not necessarily be the most efficient at maintaining subsequent growth. This emphasizes that differential effects from novel vs. local ERM partners may also have distinct costs and benefits across each life stage. ERM systems might also have different tradeoffs or effects on local adaptation than other guilds of mycorrhizae, indicating that patterns across ERM, ECM, and AM may deviate greatly from one another.

It is also possible that the increased germination rates observed on novel soil are not due to a difference in mutualist interactions, but rather a negative plant-soil feedback, possibly in the form of enemy release effects from specialized pathogens (i.e. Janzen-Connell dynamics). Both *Rhododendron* species may benefit from specialized ERM during germination and early growth, but could simultaneously contend with adverse effects from antagonistic soil biota accumulated around their local populations. If the ERM collected from local conspecific and novel con-generic soils provide similar benefits to seedling germination, but the accompanying biota in conspecific soil contained more detrimental specialized pathogens, this could result in a net zero effect on germination (Mangan et al. 2010). This could explain the unusual result in Experiment 2 where for both *Rhododendron* species there was no significant difference between their germination rates in their own conspecific local soil and in the biotic control (Fig. 3a). Additionally, if local antagonistic soil pathogens were playing a role similar in magnitude to the effects of ERM mutualisms, there would likely be a more discernible effect of conspecific local soil inoculum on seedling growth and mortality rates that was not directly paralleled with differences in ERM colonization rates. Differences in mutualistic benefits appear to be a more salient explanation, although both this and enemy release effects may occur simultaneously.

Overall, the finding that germination rates differed strongly and significantly among inoculum types for *Rhododendron* was surprising. Mycorrhizal influence over plant seed germination is common within the Orchidaceae, as their dust-like seeds are obligately mixotrophic, meaning they require the presence of suitable host fungi to trigger germination, and continued association to survive as seedlings (Malloch et al. 1980; Dearnaley 2007). Germination-triggering relationships between ERM and plant host seeds are known for some other genera within the Ericaceae, namely non-photosynthetic mycoheterotrophic Monotropoid plants (Bidartondo and Bruns, 2005). Although this phenomenon has not yet been documented in photosynthetic Ericaceae lineages, like *Rhododendron*, this provides a plausible mechanism for the significantly higher germination rates detected in our experiments. Our SEM visualizations documenting fungal hyphae contacting germinating *Rhododendron* seeds confirm that physical contact does appear to occur at this early life stage (Fig. 4c–d). However, the mechanisms by which interactions with novel ERM fungi might trigger higher germination rates than local ERM are much less clear and deserve further research.

The increased germination rates detected here when *Rhododendron* seed interacted with novel ERM communities (e.g., ~ 15% higher for *R. catawbiense*) could have substantial effects on the demography and growth of populations established in new regions. In contrast to the long-lived and stress-tolerant adults of many *Rhododendron* spp., the small seedlings that emerge from their minimally-provisioned seeds are thought to be extremely sensitive to environmental conditions and likely experience high mortality rates. Modest changes in germination and establishment rates at this critical life stage might have large effects on overall population growth rates. For example, populations of *R. ponticum* in its native range in southern Europe appear to be declining due in part to lack of seedling establishment, while those in the British Isles, where seedling establishment is common, are considered invasive (e.g., Erfmeier and Bruelheide 2004). Although we do not foresee *R. catawbiense* becoming invasive outside its native range, our results for seedling germination might suggest populations established outside the range could grow at comparable or even higher rates than those in the native range, if seedling establishment is indeed key to the species’ population dynamics.

### Insights into community composition and colonization

The increased germination of *Rhododendron* in the presence of novel mutualist partners is an important and unusual result, possibly indicating that the presence of congeners may not always limit colonization by related species, as sometimes hypothesized (e.g., Darwin’s Naturalization Hypothesis; Schaefer et al. 2011). Our results provide evidence that indirect effects of existing *Rhododendron* congeners (and the presence of their associated ERM mutualists) might actually facilitate the establishment of non-native *Rhododendron* seeds arriving in new regions via long-distance dispersal or being introduced in the context of assisted colonization. This has significant implications for incorporating biotic interactions into models of species’ range shifts, as plant × plant competition has been hypothesized to be a substantial biotic hindrance to plant range expansion (Corlett and Westcott 2013; Lockwood et al. 2013). However, given the results of these experiments, it is conceivable that the presence of native congeners in taxa that form specialized, below-ground mutualisms might ultimately provide more benefit for newly colonizing species than negative effects from interspecific competition, a dynamic that could actually favor co-existence and positive association of related species with shared mutualists (Zahra et al. 2021). Exploring this tension between the indirect benefits of shared mutualists and direct plant × plant competition would require further research pairing heterospecific individuals in shared mesocosms or experimental field plots, something that was not done in the present study. Overall, the indirect role of competitors’ associated mycorrhizae, and the possibility of congener-facilitated mutualism, has not yet been addressed in reviews that explore the impact of interspecific interactions on plant migration and colonization in response to climate change (Corlett and Westcott 2013).

### Conclusions and conservation implications

In the face of climate change, at-risk endemic plants with specialized mutualisms are of significant concern, as geographic disassociation between mutualist partners might result in lower performance and survival rates, or even extinction. Although ERM are a highly specialized form of mycorrhizae, this dynamic appears less likely than initially thought for the two *Rhododendron* species investigated here, as seeds of both species germinated better on novel ERM, indicating that high host-symbiont specificity is unlikely in this system. Instead, these results might even hint at local co-evolution and “arms races” between partners that somehow diminish the initial value of the partnership. This dynamic might underlie our results showing unexpected positive outcomes of novel partnerships for seed germination.

Overall, our results suggest that the endemic *R. catawbiense* might find suitable mutualistic ERM partners for establishment and growth poleward of its native range if its seeds arrived in these regions via natural long-distance dispersal or by intentional introduction in the context of future assisted colonization efforts. Indeed, given the findings of this study, it seems possible that this endemic species’ current absence as a native species from these northern, post-glacial regions, where climate already overlaps conditions seen in portions of the species’ native range, might trace more to seed dispersal limitation than a lack of required below-ground mutualists (cf. Bellemare and Moeller 2014; Fig. 1b) (Seliger et al. 2021). The survival of planted *R. catawbiense* in horticulture in the region, and records of occasional adventive *R. catawbiense* individuals growing near areas of human habitation in southern New England (Haines 2011), suggest aspects of its ecological niche requirements are already sometimes met in the region. However, our finding that ERM communities from the species’ native range site might provide more benefits to growing seedlings does raise the issue that any planned conservation interventions in the future, like assisted colonization, would benefit from evaluation of plant-associated soil biota, not just plants in isolation. Evidence continues to emerge that such specialized soil microbiota could be key to plant population survival (e.g., David et al. 2019). This would certainly increase the complexity of such conservation interventions, if they were deemed necessary in the future, but might better reflect the ecological reality of these species’ complex relationship to both abiotic and biotic factors.

## Supplementary Information

Below is the link to the electronic supplementary material.

Supplementary file1 (DOCX 280 KB)

## Data Availability

Upon acceptance, the data from this research project will be archived in DRUM (The Data Repository for the University of Minnesota), a publicly available database.
