# Supplementary material for: Of mutualism and migration: will interactions with novel ericoid mycorrhizal communities help or hinder northward Rhododendron range shifts?
Source: Oecologia. 2022 Jan 2;198(4):839–52. doi: 10.1007/s00442-021-05081-9 (PMC9056439; doi:10.1007/s00442-021-05081-9)
Supplement: Supplementary file 1 — Supplementary file1 (DOCX 280 KB) [file 442_2021_5081_MOESM1_ESM.docx]

**ELECTRONIC SUPPLEMENTARY MATERIAL**

Of mutualism and migration: Will interactions with novel ericoid mycorrhizal communities help or hinder northward *Rhododendron* range shifts?

Taryn L. Mueller^1*^, Elena Karlsen-Ayala^2^, David A. Moeller^3^, and Jesse Bellemare^4^

1. Department of Ecology, Evolution, & Behavior, University of Minnesota, 1479 Gortner Avenue, St. Paul, MN 55108
2. Department of Plant Pathology, University of Florida, 2550 Hull Road, Gainesville, FL 32611
3. Department of Plant and Microbial Biology, University of Minnesota, 1479 Gortner Avenue, St. Paul, MN 55108
4. Department of Biological Sciences, Smith College, 1 Chapin Way, Northampton, MA 01063

*Author for correspondence: [muellert@umn.edu](mailto:muellert@umn.edu)

**Appendix 1**

**Experiment 1B: Effect of two soil inocula on *Rhododendron* seed germination**

**Methods**

In Spring 2017, we performed an experiment to further examine the effect of inoculum from *R. maximum* soils (versus a biotic, rather than sterilized, control) on the germination of *R. maximum* and *R. catawbiense* seeds. In Fall 2016, *R. maximum* seeds were collected from multiple wild plants in a population in Shelburne, MA; *R. catawbiense* seeds were collected from a large horticultural specimen (1062PA*C) in the Smith College Botanic Garden in Northampton, MA. All seeds collected for a species were combined, mixed, and randomly subsampled for dry sowing. During seed collection in the *R. maximum* population, soil inoculum (= soil organic horizon material and fine roots to a depth of 10 cm) was also collected from beneath ten *R. maximum* plants. This inoculum was expected to contain native ericoid mycorrhizal fungi (ERM) associated with the fine roots of *R. maximum* at the site, as well as a range of other soil microbiota typical of forest soils. These soil samples were stored at 3-4°C until late February 2017, then pooled, homogenized, and subsampled for inoculations.

In addition to the targeted *R. maximum* soil inoculum described above, a second set of soil organic layer was collected in November 2016 from a conifer-dominated forest site lacking Ericaceae at Smith College’s MacLeish Field Station in Whately, MA. Due to the absence of Ericaceae in the area sampled, this organic layer was presumed to lack high abundances of ERM, but likely still contained a diverse assemblage of typical non-ERM fungi and microbes. We elected to use this organic layer material (hereafter “forest soil”) instead of standard commercially-available peat moss, since the latter is harvested from northern peatland environments that are typically dominated by Ericaceae and might be expected to contain ERM. The forest soil was refrigerated at 3-4°C until late February 2017, then homogenized and combined with washed, coarse sand (Quikcrete All-Purpose Sand no. 1152) in a 1:1 ratio to form the base potting material for the experiment.

Eighty replicate mesocosms were created using 946 mL plastic Ziploc containers with lids, each with six drainage holes drilled in the bottom and the central 6 x 6 cm of the plastic lid removed to enhance air flow and light penetration. These containers were partially filled with 500 ml of the base soil potting mix, and placed on a greenhouse bench in temperatures ranging from 23-26°C. The mesocosms were watered on a regular basis to maintain moist conditions and allowed to establish for three weeks to encourage development of fungal and microbial soil communities. After three weeks, ~ 60 cm^3^ of the homogenized *R. maximum* inoculum was added to the soil surface of half of the mesocosms (n = 40). All the mesocosms were then watered to capacity and allowed to drain, encouraging infiltration of the inoculum into the soil profile.

After another three weeks, 20 *R. maximum* seeds were sown into each of 20 mesocosms previously treated with the *R. maximum* inoculum and into each of 20 control mesocosms containing only the base potting material (n = 40 mesocosms with *R. maximum* seed). In parallel, 20 seeds of *R. catawbiense* were sown into each of another 20 inoculated and 20 control mesocosms (n = 40 mesocosms with *R. catawbiense* seed). Seed sowing involved sprinkling sets of 20 seeds onto the soil surface and lightly pressing them into place.

The mesocosms were then exposed to a 12-hour light/dark cycle of natural light and supplemental greenhouse overhead lamp light. For the first month, mesocosms were covered with clear plastic wrap to maintain high humidity, with holes punctured through the film to allow airflow. The mesocosms were watered periodically to maintain evenly moist soil conditions.

In order to mimic natural *Rhododendron* germination conditions – typically on nutrient-poor, acidic soils with a carpet of low-growing bryophytes (J. Bellemare, pers. obs.) – the mesocosms were not fertilized and bryophytes were allowed to establish from the natural soil “spore bank.” As the experiment progressed, taller moss gametophytes (e.g., *Polytrichum* spp.) were trimmed to prevent overtopping of the small *Rhododendron* seedlings and any non-*Rhododendron* vascular plant seedlings that emerged were removed.

The mesocosms were surveyed during weeks 4-5 after seed sowing for evidence of *Rhododendron* seed germination based on visible emergence of radicles and/or cotyledons. The total number of seeds germinated was scored for each mesocosm, with any seedlings of uncertain identification left uncounted until they grew sufficiently large to be positively identified. The final tally of total germinants at five weeks was used in the statistical analyses presented here.

For Experiment 1B, we tested for the effects of two soil inocula (*R. maximum* soil vs. biotic control), plant species identity (*R. maximum* vs. *R. catawbiense*), and their interaction on seed germination using a generalized linear binomial model with a logit link.

We conducted binomial models using the [glm] package in R (RStudio Version 1.1.453; RStudio Team 2018).

**Results:**

Inoculum treatment (*P* < 0.001) and treatment/species interaction (*P* < 0.001) were highly significant predictors of germination rate (Fig. 1). Germination rate of *R. catawbiense* seed was significantly higher in mesocosms with the novel *R. maximum* inoculum (75.7% ± 5.12% SE) than in mesocosms with the forest soil biotic control (45.3% ± 3.41% SE, *P* < 0.001) (Fig. 1). Germination rate for *R. maximum* seed was not significantly different between the inoculum treatment (63.3% ± 4.27% SE) and the control (55.5%, ± 4.78% SE, *P* = 0.200). Some differences in germination rate were observed between the two species within the same treatment. (Fig. 1).

**
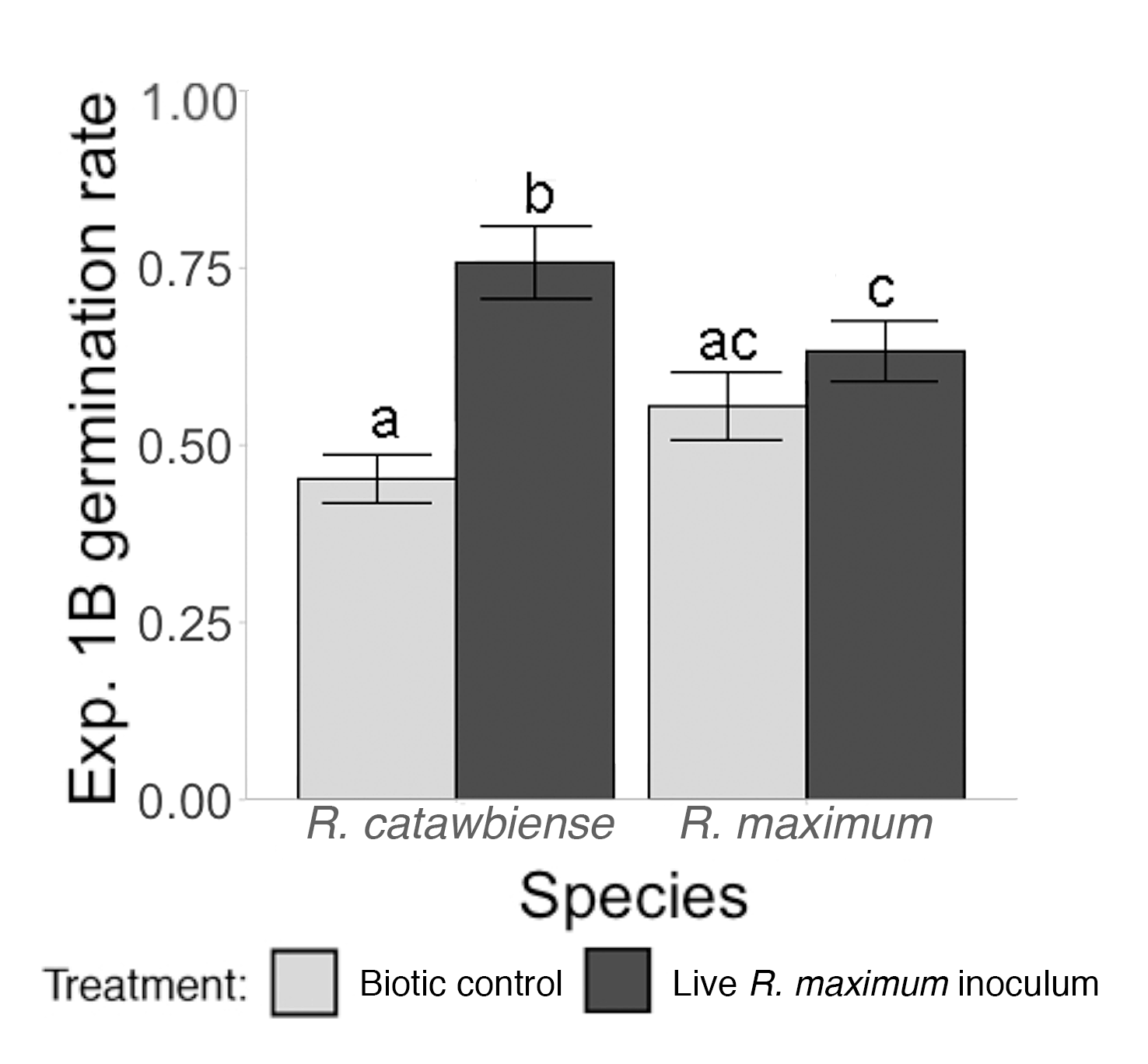
**

**Fig. 1** Germination rates of *R. maximum* and *R. catawbiense* in Experiment 1B when seeds were exposed to soil inoculated with *R. maximum*soil organic material vs. control. Significant Tukey's test differences with Bonferroni-Holm correction (*P* < 0.05) among categories within each sub-panel are indicated with differing letters

**Appendix 2. Summary statistics and post-hoc Tukey comparisons for all experiments.**

In Experiment 1, inoculum treatment (*P* < 0.00001), species (*P* = 0.0058), and treatment/species interaction (*P* < 0.0033) were highly significant predictors of germination rate (Fig. 2a).

Table 2.1: Germination rate summary statistics for each species/treatment combination in Experiment 1, showing average germination rate (GR), standard error (SE), and sample size (N). Post-hoc Tukey HSD significance values are shown for 4 pre-planned pairwise comparisons. P-values are shown as (raw p-value, Holm-corrected p-value) with significance denoted by <0.05 *, <0.01**, and <0.001***.

| Seedling species  (soil treatment) | GR | SE | N | *R. catawbiense* (Sterile control) | *R. catawbiense* (*R. maximum inoculum*) | *R. maximum* (Sterile control) |
| --- | --- | --- | --- | --- | --- | --- |
| *R. catawbiense* (Sterile control) | 0.545 | 0.047 | 15 |  |  |  |
| *R. catawbiense* (*R. maximum inoculum*) | 0.752 | 0.036 | 15 | <0.001, <0.001*** |  |  |
| *R. maximum* (Sterile control) | 0.544 | 0.039 | 15 | 0.95, 0.95 | N/A |  |
| *R. maximum*  (*R. maximum inoculum*) | 0.657 | 0.049 | 15 | N/A | <0.001, <0.001*** | <0.001, <0.001*** |

Table 2.2: Germination rate summary statistics for each species/treatment combination in Experiment 1B, showing average germination rate (GR), standard error (SE), and sample size (N). Post-hoc Tukey HSD significance values are shown for 4 pre-planned pairwise comparisons. P-values are shown as (raw p-value, Holm-corrected p-value) with significance denoted by <0.05 *, <0.01**, and <0.001***.

| Seedling species  (soil treatment) | GR | SE | N | *R. catawbiense* (Biotic control) | *R. catawbiense* (*R. maximum inoculum*) | *R. maximum* (biotic control) |
| --- | --- | --- | --- | --- | --- | --- |
| *R. catawbiense* (Biotic control) | 0.453 | 0.034 | 20 |  |  |  |
| *R. catawbiense* (*R. maximum inoculum*) | 0.758 | 0.051 | 20 | <0.001, <0.001*** |  |  |
| *R. maximum* (Biotic control) | 0.555 | 0.048 | 20 | 0.041, 0.082 | N/A |  |
| *R. maximum*  (*R. maximum inoculum*) | 0.633 | 0.043 | 20 | N/A | 0.008, 0.024  * | 0.200, 0.200 |

In Experiment 1, average largest leaf size was influenced significantly only by soil treatment (df =1, χ^2^= 27.674 , *P* < 0.0001)

Table 2.3: Largest leaf size summary statistics for each species/treatment combination in Experiment 1, showing average largest leaf size in mm (LLS), standard error (SE), and sample size (N). Post-hoc Tukey HSD significance values are shown for 4 pre-planned pairwise comparisons. P-values are shown as (raw p-value, Holm-corrected p-value) with significance denoted by <0.05 *, <0.01**, and <0.001***.

| Seedling species  (soil treatment) | LLS (mm) | SE | N | *R. catawbiense* (Sterile control) | *R. catawbiense* (*R. maximum inoculum*) | *R. maximum* (Sterile control) |
| --- | --- | --- | --- | --- | --- | --- |
| *R. catawbiense* (Sterile control) | 6.24 | 2.12 | 75 |  |  |  |
| *R. catawbiense* (*R. maximum inoculum*) | 4.08 | 1.25 | 75 | 0.002, 0.006 *** |  |  |
| *R. maximum* (Sterile control) | 6.00 | 1.89 | 75 | 0.730, 0.690 | N/A |  |
| *R. maximum*  (*R. maximum inoculum*) | 3.45 | 1.23 | 75 | N/A | 0.160, 0.310 | <0.001, <0.001*** |

For Experiment 2, average largest leaf size (hereafter LLS) was influenced significantly by soil treatment (df =1, χ^2^= 27.674 , *P* < 0.0001)

Table 2.4: Germination rate summary statistics for each species/treatment combination in Experiment 2, showing average germination rate (GR), standard error (SE), and sample size (N). Post-hoc Tukey HSD significance values are shown for 9 pre-planned pairwise comparisons. P-values are shown as (raw p-value, Holm-corrected p-value) with significance denoted by <0.05 *, <0.01**, and <0.001***.

| Seedling species  (soil treatment) | GR | SE | N | *R. catawbiense* (Control) | *R. catawbiense* (*R. maximum*) | *R.*  *catawbiense* (*R. catawbiense*) | *R.*  *maximum* (Control) | *R.*  *maximum*  (*R. maximum*) |
| --- | --- | --- | --- | --- | --- | --- | --- | --- |
| *R. catawbiense* (Control) | 0.516 | 0.030 | 25 |  |  |  |  |  |
| *R. catawbiense* (*R. maximum*) | 0.64 | 0.033 | 25 | <0.001, <0.001*** |  |  |  |  |
| *R. catawbiense* (*R. catawbiense*) | 0.492 | 0.023 | 25 | 0.44, 0.95 | <0.001, <0.001  *** |  |  |  |
| *R. maximum* (Control) | 0.646 | 0.037 | 25 | <0.001, <0.001*** | N/A | N/A |  |  |
| *R. maximum*  (*R. maximum*) | 0.67 | 0.025 | 25 | N/A | 0.310, 0.950 | N/A | 0.420, 0.950 |  |
| *R. maximum*  (*R. catawbiense*) | 0.776 | 0.019 | 25 | N/A | N/A | <0.001, <0.001  *** | <0.001, <0.001  *** | 0.002, 0.011 * |

For Experiment 2, LLS was influenced significantly by soil inoculum (df =2, χ^2^ =18.7, *P* < 0.0001).

Table 2.5: Largest leaf size summary statistics for each species/treatment combination in Experiment 2, showing average largest leaf size in mm (LLS), standard error (SE), and sample size (N). Post-hoc Tukey HSD significance values are shown for 9 pre-planned pairwise comparisons. P-values are shown as (raw p-value, Holm-corrected p-value) with significance denoted by <0.05 *, <0.01**, and <0.001***.

| Seedling species  (soil treatment) | LLS  (mm) | SE | N | *R. catawbiense* (Control) | *R. catawbiense* (*R. maximum*) | *R. catawbiense* (*R. catawbiense*) | *R. maximum* (Control) | *R. maximum*  (*R. maximum*) |
| --- | --- | --- | --- | --- | --- | --- | --- | --- |
| *R. catawbiense* (Control) | 4.31 | 0.167 | 125 |  |  |  |  |  |
| *R. catawbiense* (*R. maximum*) | 4.66 | 0.215 | 125 | 0.910, 1.00 |  |  |  |  |
| *R. catawbiense* (*R. catawbiense*) | 5.16 | 0.199 | 125 | 0.210, 1.00 | 0.400, 1.00 |  |  |  |
| *R. maximum* (Control) | 3.32 | 0.122 | 125 | 0.008, 0.068 | N/A | N/A |  |  |
| *R. maximum*  (*R. maximum*) | 4.70 | 0.220 | 125 | N/A | 0.940, 1.00 | N/A | 0.008, 0.068 |  |
| *R. maximum*  (*R. catawbiense*) | 5.06 | 0.159 | 125 | N/A | N/A | 0.94, 1.000 | <0.001, <0.001  *** | 0.022, 0.110 |

For Experiment 2, species identity had a much greater effect on seedling mortality rates than soil inoculum type or presence, as species (df =1, χ^2^ = 32.7, *P* < 0.0001) and inoculum treatment/species interaction (df =2, χ^2^ = 9.61, *P* = 0.008) were significant predictors of mortality rate, while inoculum treatment alone was not significant (df =2, χ^2^ = 0.77, *P* = 0.68).

Table 2.6: Survival rate summary statistics for each species/treatment combination in Experiment 2, showing average mortality rate (MR), standard error (SE), and sample size (N). Post-hoc Tukey HSD significance values are shown for 9 pre-planned pairwise comparisons. P-values are shown as (raw p-value, Holm-corrected p-value) with significance denoted by <0.05 *, <0.01**, and <0.001***.

| Seedling species  (soil treatment) | MR | SE | N | *R. catawbiense* (Control) | *R. catawbiense* (*R. maximum*) | *R.*  *catawbiense* (*R. catawbiense*) | *R.*  *maximum* (Control) | *R.*  *maximum*  (*R. maximum*) |
| --- | --- | --- | --- | --- | --- | --- | --- | --- |
| *R. catawbiense* (Control) | 0.076 | 0.015 | 25 |  |  |  |  |  |
| *R. catawbiense* (*R. maximum*) | 0.09 | 0.027 | 25 | 0.420, 0.990 |  |  |  |  |
| *R. catawbiense* (*R. catawbiense*) | 0.058 | 0.011 | 25 | 0.250, 0.990 | 0.055, 0.330 |  |  |  |
| *R. maximum* (Control) | 0.136 | 0.030 | 25 | 0.002, 0.018  * | N/A | N/A |  |  |
| *R. maximum*  (*R. maximum*) | 0.12 | 0.025 | 25 | N/A | 0.240, 0.990 | N/A | 0.250, 0.990 |  |
| *R. maximum*  (*R. catawbiense*) | 0.168 | 0.024 | 25 | N/A | N/A | <0.001, <0.001  *** | 0.150, 0.790 | 0.011, 0.078 |

For Experiment 2, in the analysis of ERM seedling root colonization rates, the only significant model term was soil treatment (df =2, F = 145.4, *P* < 0.0001).

Table 2.7: Mycorrhizal colonization rate summary statistics for each species/treatment combination in Experiment 2, showing average percent colonization (PC), standard error (SE), and sample size (N). Post-hoc Tukey HSD significance values are shown for 9 pre-planned pairwise comparisons. P-values are shown as (raw p-value, Holm-corrected p-value) with significance denoted by <0.05 *, <0.01**, and <0.001***.

| Seedling species  (soil treatment) | PC  (%) | SE | N | *R. catawbiense* (Control) | *R. catawbiense* (*R. maximum*) | *R.*  *catawbiense* (*R. catawbiense*) | *R.*  *maximum* (Control) | *R.*  *maximum*  (*R. maximum*) |
| --- | --- | --- | --- | --- | --- | --- | --- | --- |
| *R. catawbiense* (Control) | 3.27 | 1.04 | 75 |  |  |  |  |  |
| *R. catawbiense* (*R. maximum*) | 20.83 | 2.49 | 75 | <0.001, <0.001*** |  |  |  |  |
| *R. catawbiense* (*R. catawbiense*) | 24.39 | 1.84 | 75 | <0.001, <0.001*** | 0.007, 0.028  * |  |  |  |
| *R. maximum* (Control) | 5.18 | 1.19 | 75 | 0.180, 0.550 | N/A | N/A |  |  |
| *R. maximum*  (*R. maximum*) | 19.03 | 2.27 | 75 | N/A | 0.620, 0.990 | N/A | <0.001, <0.001*** |  |
| *R. maximum*  (*R. catawbiense*) | 28.56 | 2.17 | 75 | N/A | N/A | 0.500, 0.990 | <0.001, <0.001  *** | <0.001,  <0.001*** |
